# Supplementary material for: Refining the impact of genetic evidence on clinical success
Source: Nature. 2024 Apr 17;629(8012):624–9. doi: 10.1038/s41586-024-07316-0 (PMC11096124; doi:10.1038/s41586-024-07316-0)
Supplement: Supplementary file 1 — Supplementary Figs. 1–9, corresponding to the three main and six extended data figures restricted to drugs with one target only. [file 41586_2024_7316_MOESM1_ESM.pdf]

nature portfolio

https://doi.org/10.1038/s41586-024-07316-0

Supplementary information

Refining the impact of genetic evidence on clinical success

In the format provided by the authors and unedited

SUPPLEMENT

Refining the impact of genetic evidence on clinical success

Eric Vallabh Minikel, Jeffery L Painter, Coco Chengliang Dong, Matthew R. Nelson

The supplementary figures below correspond to main and extended data figures, with Pharmaprojects filtered for only those drugs with a single target assigned ("one target only" mode). Supplementary tables are provided in a separate Excel file.

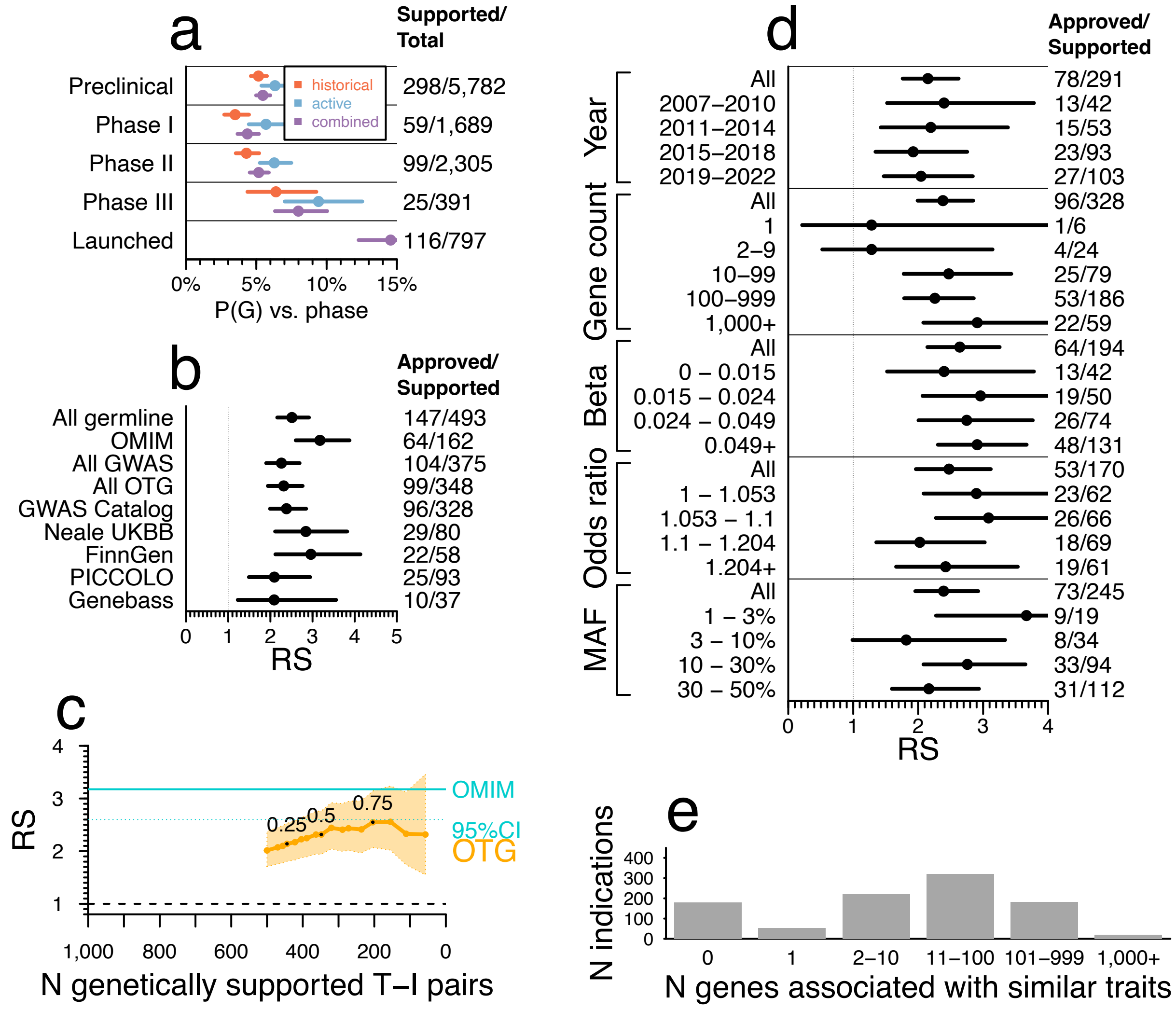

Figure S1. Impact of genetic evidence characteristics on relative success (Figure 1) in "one target only" mode. A) *Proportion of target-indication (T-I) pairs with genetic support, P(G), as a function of highest phase reached. The center is the exact proportion and error bars are Wilson 95% confidence intervals. The N is indicated at right, with the denominator being the total number of T-I pairs reaching each phase, and the numerator being the number of those that are genetically supported.* ***B)*** *Sensitivity of relative success (RS) from phase I–launch of T-I pairs with genetic evidence to source of human genetic association. GWAS Catalog, Neale UKBB, and FinnGen are subsets of Open Targets Genetics (OTG). The center is the point estimate of RS (see Methods) and bars are Katz 95% confidence intervals. The N is indicated at right, with the denominator being the number of T-I pairs with genetic support from each source, and the numerator being the number that are launched. Note that RS is calculated from a 2x2 contingency table and is also dependent upon the number of T-I pairs without genetic support. Total N = 7,870 T-I pairs.* ***C)*** *Sensitivity of RS to locus-to-gene (L2G) share threshold among OTG genome-wide association study (GWAS) significant associations. The*

minimum L2G share required for inclusion in the dataset is varied from 0.1 to 1.0 in increments of 0.05 (labels) while RS (y axis) is plotted against the number of clinical (phase I+) programs considered to have genetic support from OTG (x axis). The line represents point estimates of RS as a function of L2G threshold while shaded areas are Katz 95% confidence intervals. ***D)*** *Sensitivity of RS for OTG GWAS-supported T-I pairs to binned variables: i) year in which a T-I pair first acquired human genetic support from GWAS, excluding replications and excluding T-I pairs otherwise supported by OMIM, ii) number of genes exhibiting genetic association to the same trait, iii) quartile of effect size (beta) for quantitative traits, iv) quartile of effect size (odds ratio, OR) for case/control traits standardized to be >1 (i.e., 1/OR if <1), and v) order of magnitude of minor allele frequency bins. The center is the point estimate of RS (see Methods) and bars are Katz 95% confidence intervals. The N is indicated at right, as in (B). Total N = 7,870 T-I pairs.* ***E)*** *Count of indications ever in development in Pharmaprojects (y axis) by the number of genes associated with traits similar to those indications (x axis).*

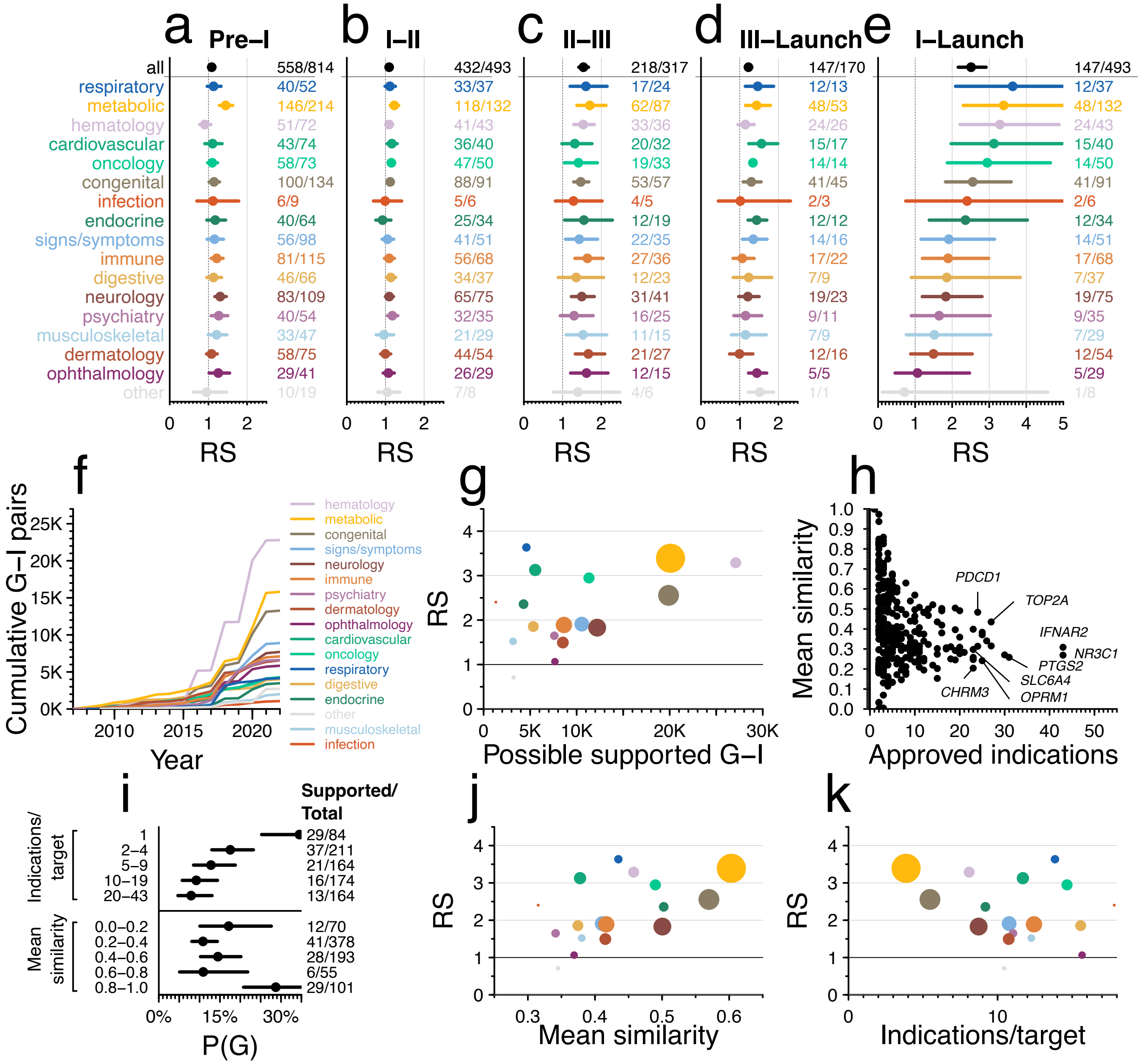

Figure S2. Differences in relative success between therapy areas and the number and diversity of indications per target (Figure 2) in "one target only" mode. A-E) *RS by therapy area and phase transition. The center is the point estimate of RS (see Methods) and bars are 95% confidence intervals. The N is indicated at right, with the denominator being the number of T-I pairs with genetic support, and the numerator being that succeeded in the phase transition indicated at the top of the panel. Note that RS is calculated from a 2x2 contingency table and is also dependent upon the number of T-I pairs without genetic support. For "all", total N=14,307 preclinical, 7,870 reaching at least phase I, 4,249 reaching at least phase II, and 1,413 reaching at least phase III.* ***F****) Cumulative number of possible genetically supported gene-indication (G-I) pairs in each therapy (y axis) as genetic discoveries have accrued over time (x axis).* ***G****) RS (y axis) by number of possible supported G-I pairs (x axis) across therapy areas, dots colored as in panels A-E and sized according to number of genetically supported T-I pairs in at least phase I.* ***H)*** *Number of launched indications vs. similarity of those indications, by approved drug target.* ***I)*** *Proportion of launched target-indication pairs with genetic support, P(G), binned by quintile of the number of launched indications per target (top panel) or by mean similarity among launched indications (bottom panel). Targets with exactly 1 launched indication (15.3% of launched T-I pairs) are considered to have mean similarity of 1.0. The center is the exact proportion and bars*

are Wilson 95% confidence intervals. The N is indicated at right, where the denominator is the total number of launched T-I pairs in each bin and the numerator is the number of those that are genetically supported. ***J)*** *RS (y axis) vs. mean similarity among launched indications per target (x axis) by therapy area.* ***K)*** *RS (y axis) vs. mean count of launched indications per target (x axis).*

a

	all genes	predicted Ab tractable	predicted SM tractable	rhodopsin–like GPCRs	nuclear receptors	enzymes	ion channels	kinases
all areas	478/34,190 1.4%	395/17,811 2.2%	394/12,391 3.2%	24/508 4.7%	16/245 6.5%	71/2,278 3.1%	21/714 2.9%	67/1,134 5.9%
respiratory	21/884 2.4%	20/480 4.2%	11/318 3.5%	2/9 22%	0/5 0%	1/51 2%	1/26 3.8%	0/13 0%
metabolic	92/6,819 1.3%	76/3,594 2.1%	75/2,487 3%	5/90 5.6%	6/51 12%	10/471 2.1%	1/98 1%	3/185 1.6%
hematology	29/4,794 0.6%	26/2,409 1.1%	24/1,698 1.4%	0/87 0%	0/30 0%	6/276 2.2%	0/58 0%	1/156 0.64%
cardiovascular	28/2,681 1%	28/1,436 1.9%	25/952 2.6%	1/43 2.3%	0/17 0%	9/212 4.2%	6/97 6.2%	0/90 0%
oncology	168/4,304 3.9%	130/2,088 6.2%	158/1,975 8%	5/37 14%	4/25 16%	33/419 7.9%	0/56 0%	52/250 21%
congenital	75/4,488 1.7%	58/2,444 2.4%	54/1,697 3.2%	1/46 2.2%	1/28 3.6%	13/296 4.4%	1/103 0.97%	2/114 1.8%
infection	3/234 1.3%	3/138 2.2%	1/88 1.1%	0/4 0%	0/2 0%	0/13 0%	0/7 0%	0/2 0%
endocrine	27/2,676 1%	23/1,327 1.7%	24/922 2.6%	5/53 9.4%	2/32 6.2%	1/187 0.53%	1/53 1.9%	2/80 2.5%
signs/symptoms	31/3,629 0.85%	27/1,897 1.4%	27/1,200 2.2%	3/70 4.3%	2/30 6.7%	3/206 1.5%	8/124 6.5%	3/107 2.8%
immune	50/2,406 2.1%	46/1,301 3.5%	26/855 3%	2/40 5%	0/7 0%	7/125 5.6%	0/26 0%	2/79 2.5%
digestive	24/2,309 1%	18/1,252 1.4%	14/802 1.7%	0/47 0%	2/21 9.5%	2/129 1.6%	1/24 4.2%	2/71 2.8%
neurology	60/3,481 1.7%	51/1,911 2.7%	45/1,212 3.7%	2/53 3.8%	0/13 0%	8/198 4%	9/133 6.8%	2/86 2.3%
psychiatry	23/2,382 0.97%	21/1,300 1.6%	22/786 2.8%	5/53 9.4%	0/18 0%	3/131 2.3%	4/84 4.8%	0/74 0%
musculoskeletal	23/1,620 1.4%	22/887 2.5%	17/539 3.2%	1/15 6.7%	1/6 17%	1/91 1.1%	1/31 3.2%	3/54 5.6%
dermatology	36/2,217 1.6%	31/1,183 2.6%	23/731 3.1%	2/24 8.3%	0/6 0%	6/131 4.6%	0/28 0%	3/71 4.2%
ophthalmology	21/2,044 1%	16/1,153 1.4%	13/626 2.1%	1/19 5.3%	1/18 5.6%	2/108 1.9%	2/54 3.7%	0/61 0%
other	5/1,513 0.33%	4/761 0.53%	3/487 0.62%	0/24 0%	1/11 9.1%	0/93 0%	0/35 0%	0/41 0%

b

	all genes	predicted Ab tractable	predicted SM tractable	rhodopsin–like GPCRs	nuclear receptors	enzymes	ion channels	kinases
oncogene	38/184 21%	32/94 34%	36/118 31%	1/3 33%	1/2 50%	7/18 39%	0/1 0%	14/25 56%
tumor suppressor	7/197 3.6%	2/69 2.9%	5/103 4.9%	0/1 0%	0/1 0%	3/25 12%	0/0 –	0/10 0%
unknown	12/208 5.8%	10/102 9.8%	10/96 10%	1/2 50%	0/0 –	1/13 7.7%	0/1 0%	1/18 5.6%

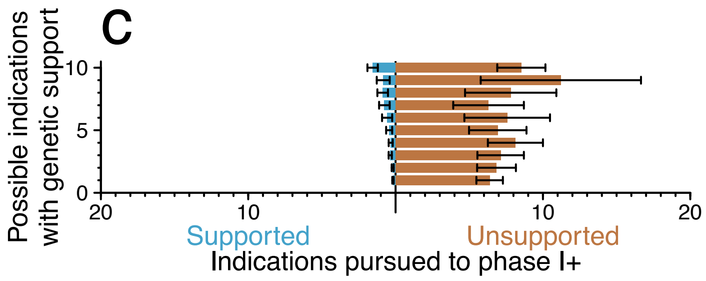

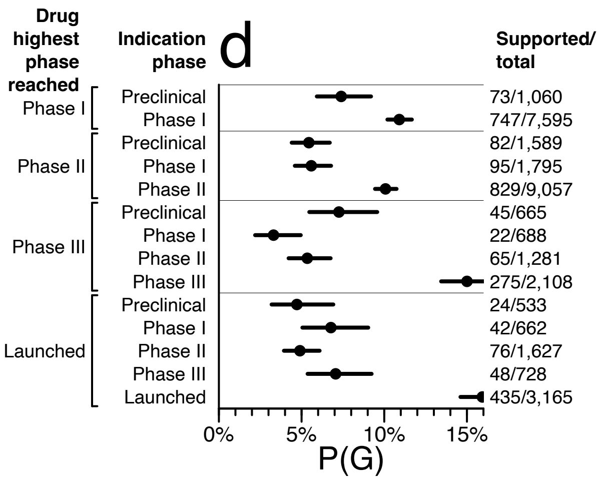

Figure S3. Clinical investigation of drug mechanisms with genetic evidence (Figure 3) in "one target only" mode. A) *Heatmap of proportion of genetically supported T-I pairs that have been developed to at least phase I, by therapy area (y axis) and gene list (x axis).* ***B)*** *As panel A, but for genetic support from IntOGen rather than germline sources and grouped by the direction of effect of the gene according to IntOGen (y axis), and also grouped by target rather than T-I pair. Thus, the denominator for each cell is the number of targets with at least one genetically supported indication, and each target counts towards the numerator if at least one genetically supported indication has reached phase I.* ***C)*** *Of targets that have both reached phase I for any indication, and have at least one genetically supported indication, the mean count (x axis) of genetically supported (left) and unsupported (right) indications pursued, binned by the number of possible genetically supported indications (y axis). The center is the mean and bars are Wilson 95% confidence intervals. N = 937 targets.* ***D)*** *Proportion of D-I pairs with genetic support, P(G) (x axis), as a function of each D-I pair's phase reached (inner y axis grouping) and the drug's highest phase reached for any indication (outer y axis grouping). The center is the exact proportion and bars are Wilson 95% confidence intervals. The N is indicated*

at right, where the denominator is the total number of D-I pairs in each bin, and the numerator is the number of those that are genetically supported.

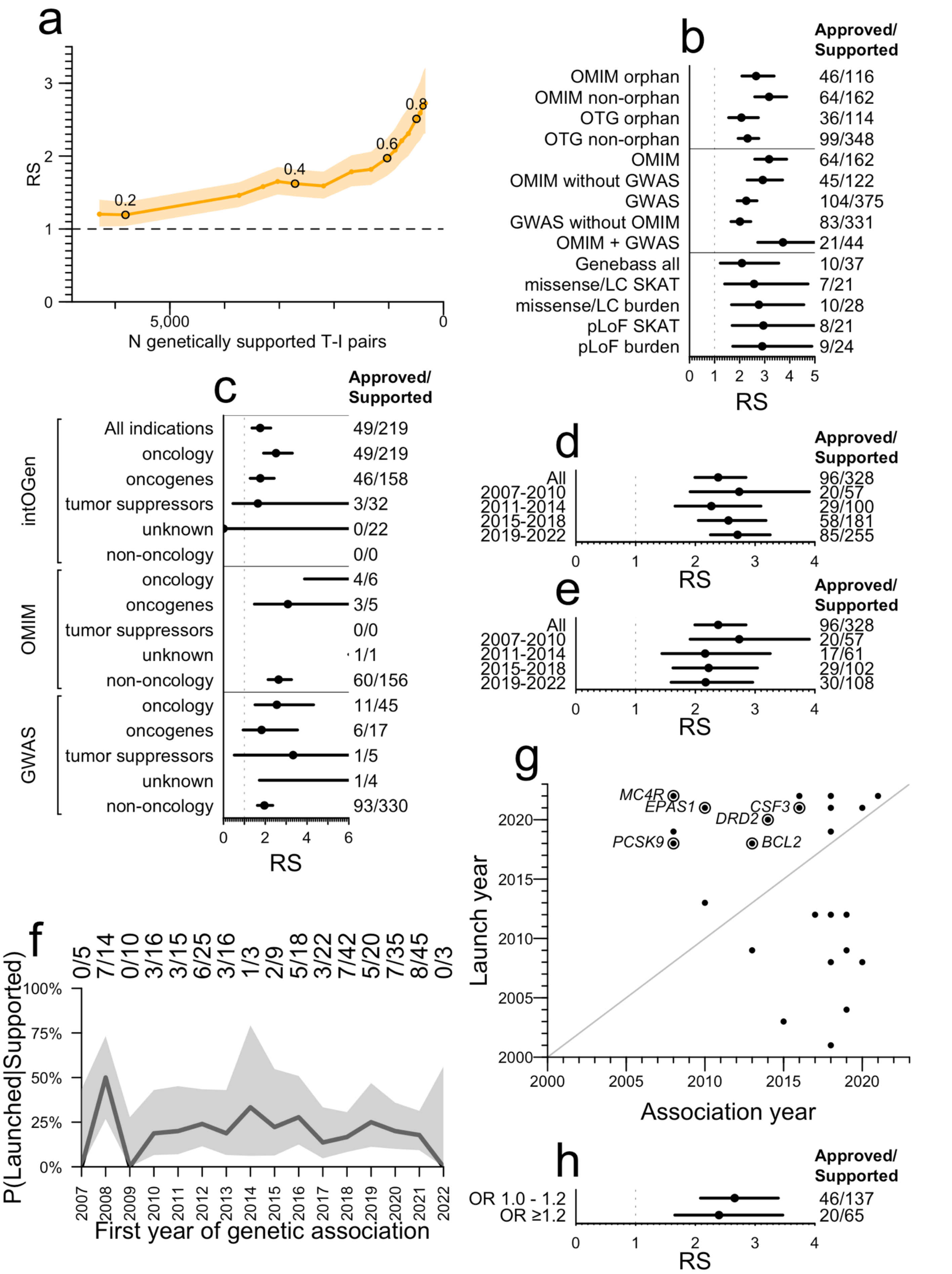
a
b
c
d
e
f
g
h
RS
N genetically supported T-I pairs
Approved/ Supported
OMIM orphan 46/116
OMIM non-orphan 64/162
OTG orphan 36/114
OTG non-orphan 99/348
OMIM 64/162
OMIM without GWAS 45/122
GWAS 104/375
GWAS without OMIM 83/331
OMIM + GWAS 21/44
Genebass all 10/37
missense/LC SKAT 7/21
missense/LC burden 10/28
pLoF SKAT 8/21
pLoF burden 9/24
intOGen
All indications 49/219
oncology 49/219
oncogenes 46/158
tumor suppressors 3/32
unknown 0/22
non-oncology 0/0
OMIM
oncology 4/6
oncogenes 3/5
tumor suppressors 0/0
unknown 1/1
non-oncology 60/156
GWAS
oncology 11/45
oncogenes 6/17
tumor suppressors 1/5
unknown 1/4
non-oncology 93/330
All 96/328
2007-2010 20/57
2011-2014 29/100
2015-2018 58/181
2019-2022 85/255
All 96/328
2007-2010 20/57
2011-2014 17/61
2015-2018 29/102
2019-2022 30/108
P(Launched|Supported)
First year of genetic association
0/5 7/14 0/10 3/16 3/15 6/25 3/16 1/3 2/9 5/18 3/22 7/42 5/20 7/35 8/45 0/3
Launch year
Association year
MC4R
EPAS1
CSF3
DRD2
PCSK9
BCL2
OR 1.0 - 1.2 46/137
OR ≥1.2 20/65

Figure S4. Further analysis of influence of characteristics of genetic associations on relative success (Extended Data Figure 2) in "one target only" mode. A) *Sensitivity of RS to the similarity threshold between the MeSH ID for the genetically associated trait and the MeSH ID for the clinically developed indication. The threshold is varied by units of 0.05 (labels) and the results are plotted as RS (y axis) versus number of genetically supported T-I pairs (x axis).* ***B)*** *Breakdown of OTG and OMIM RS values by whether any drug for each T-I pair has had orphan status assigned. The N of genetically supported T-I pairs (denominator) and, of those, launched T-I pairs (numerator) is shown at right. Total N = 8,780 T-I pairs, of which 3,149 are orphan. The center is the RS point estimate and error bars are Katz 95% confidence intervals.* ***C)*** *RS for somatic genetic evidence from IntOGen versus germline genetic evidence, for oncology and non-oncology indications. Note that the approved/supported proportions displayed for the top two rows are identical because all IntOGen genetic support is for oncology indications, yet the RS is different because the number of non-supported approved and non-supported clinical stage programs is different. In other words, in the "All indications" row, there is a Simpson's paradox that diminishes the apparent RS of IntOGen — IntOGen support improves success rate (see 2nd row) but also selects for oncology, an area with low baseline success rate (as shown in Extended Data Fig. 6A). N is displayed at right as in (B). Total N = 8,780 T-I pairs, of which 4,504 non-oncology, 3,366 oncology, 606 targeting IntOGen oncogenes, 155 targeting tumor suppressors, and 95 targeting IntOGen genes of unknown mechanism. The center is the RS point estimate and error bars are Katz 95% confidence intervals.* ***D)*** *As for top panel of Figure 1D, but without removing replications or OMIM-supported T-I pairs. N is displayed as in (B). Total N = 8,780 T-I pairs. The center is the RS point estimate and error bars are Katz 95% confidence intervals.* ***E)*** *As for top panel of Figure 1D, removing replications but not removing OMIM-supported T-I pairs. N is displayed as in (B). Total N = 8,780 T-I pairs. The center is the RS point estimate and error bars are Katz 95% confidence intervals.* ***F)*** *Proportion of T-I pairs supported by a GWAS Catalog association that are launched (versus phase I-III) as a function of the year of first genetic association.* ***G)*** *Launched T-I pairs genetically supported by OTG GWAS, shown by year of launch (y axis) and year of first genetic association (x axis). Gene symbols are labeled for first approvals of targets with at least 5 years between association and launch. Of 60 OTG-supported launched T-I pairs (Fig. 1D), year of drug launch was available for N=24 shown here, of which 10 (42%) acquired genetic support only in or after the year of launch. The true proportion of launched T-I whose GWAS support is retrospective may be larger if the T-I with a missing launch year are more often older drug approvals less well annotated in Pharmaprojects.* ***H)*** *Lack of impact of GWAS Catalog lead SNP odds ratio (OR) on RS when using the same OR breaks as used by King et al[15]. N is displayed as in (B). Total N = 8,780 T-I pairs. The center is the RS point estimate and error bars are Katz 95% confidence intervals.*

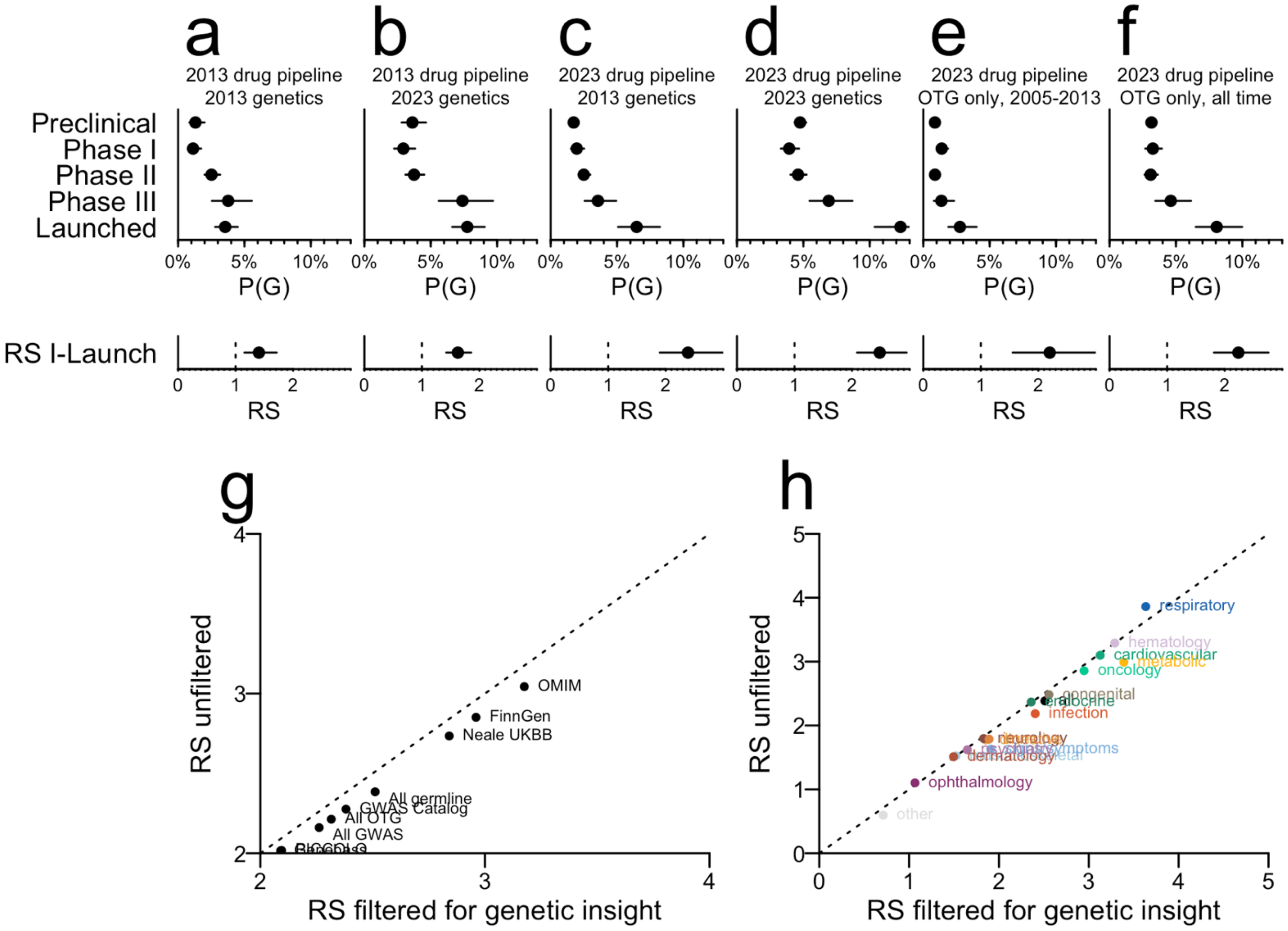

Figure S5. Sensitivity to changes in genetic data and drug pipeline over the past decade and to the 'genetic insight' filter (Extended Data Figure 3) in "one target only" mode. *"2013" here indicates the data freezes from Nelson et al 2015[5] (that study's supplementary dataset 2 for genetics and supplementary dataset 3 for drug pipeline); "2023" indicates the data freezes in the present study. All datasets were processed using the current MeSH similarity matrix, and because "genetic insight" changes over time (more traits have been studied genetically now than in 2013), all panels are unfiltered for genetic insight (hence numbers in panel D differ from those in Fig. 1A). Every panel shows the proportion of combined (both historical and active) target-indication pairs with genetic support, or P(G), by development phase. For this supplementary figure, note that "one target only" mode can only be applied to the 2023 drug pipeline dataset; 2013 drug pipeline information is simply used as-is from the Nelson 2015 publication and cannot be filtered for "one target only" drugs.* ***A)*** *2013 drug pipeline and 2013 genetics.* ***B)*** *2013 drug pipeline and 2023 genetics.* ***C)*** *2023 drug pipeline and 2013 genetics.* ***D)*** *2023 drug pipeline and 2023 genetics.* ***E)*** *2023 drug pipeline with only OTG GWAS hits through 2013 and no other sources of genetic evidence.* ***F)*** *2023 drug pipeline with only OTG GWAS hits for all years, no other sources of genetic evidence. We note that the increase in P(G) over the past decade[5] is almost entirely attributable to new genetic evidence (e.g. contrast B vs. A, D vs. C, F vs. E) rather than changes in the drug pipeline (e.g. compare A vs. C, B vs. D). In contrast, the increase in RS is due mostly to changes in the drug pipeline (compare C, D, E, F vs. A, B), in line with theoretical expectations outlined by Hingorani et al[16] and consistent with the findings of King et al[15]. We note that both the contrasts in this figure, and the fact that genetic support is so often retrospective (Extended Data Fig. 2G) suggest that P(G) will continue to rise in coming years. For 2013 drug pipeline, N=8,624 T-I pairs (1,605 preclinical, 1,772 phase I, 2,779 phase II, 636 phase III, and 1,832 launched); for 2023 drug pipeline, N=18,783 T-I pairs (N=9,132 preclinical, 3,102 phase I, 4,652 phase II, 955 phase III, and 942 launched). In A-F, the center is exact proportion and error bars are Wilson binomial 95% confidence intervals. Because all panels here are unfiltered for genetic insight, we also show the difference in RS across* ***G)*** *sources of genetic evidence and* ***H)*** *therapy areas when this filter is removed. In general, removing this filter decreases RS by 0.13; this varies only slightly between sources and*

areas. The largest impact is seen in "signs/symptoms", where removing the filter drops the RS from 1.91 to 1.63. The relatively minor impact of removing the genetic insight filter is consistent with the findings of King et al[15]*, who varied the minimum number of genetic associations required for an indication to be included, and found that risk ratio for progression (i.e. RS) was slightly diminished when the threshold was reduced.*

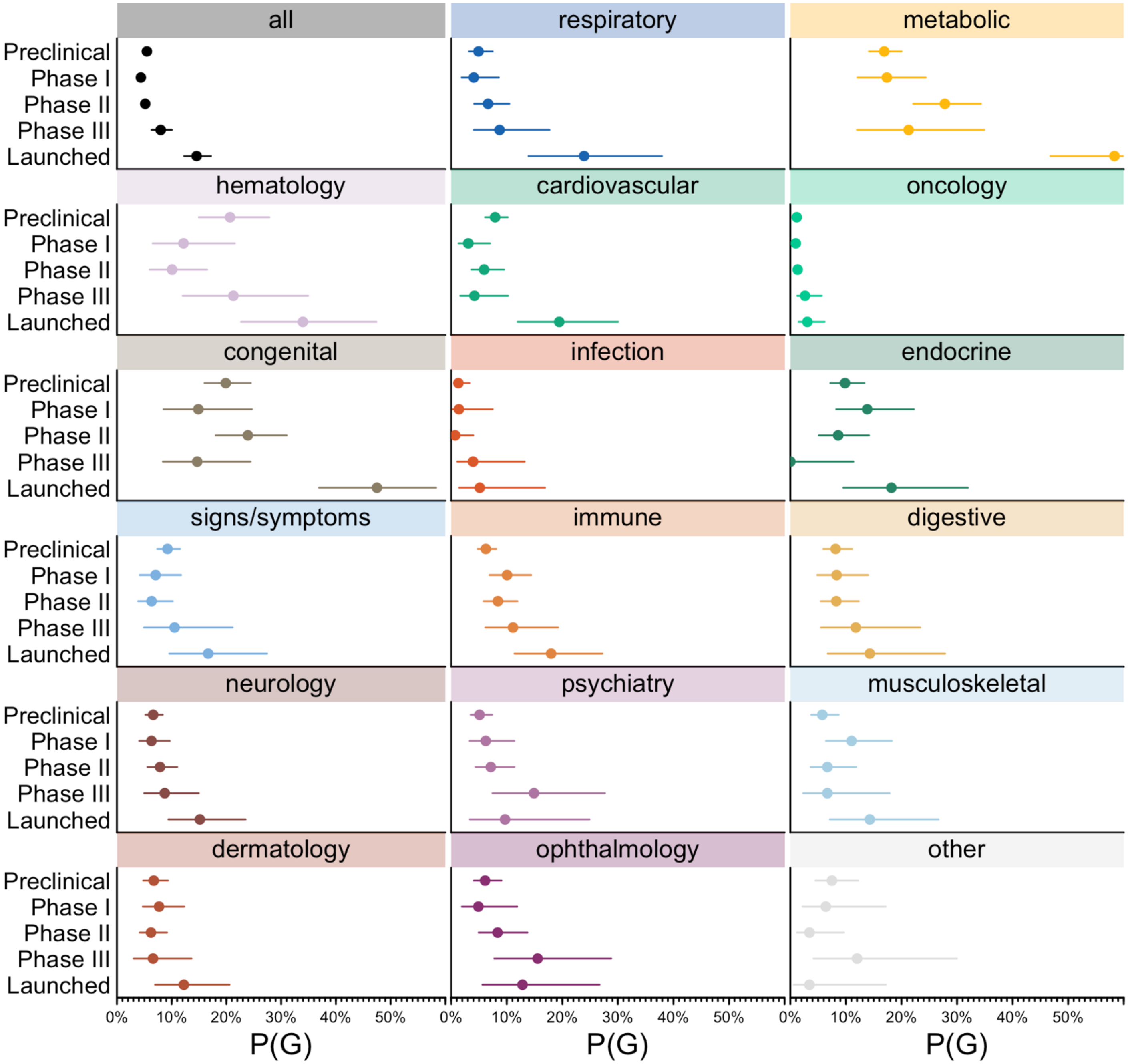

Figure S6. P(G) by phase versus therapy area (Extended Data Figure 5) in "one target only" mode. *Each panel represents one therapy area, and shows the proportion of target-indication pairs in that area with genetic support, or P(G), by development phase. Total number of T-I pairs in any area: N=7,884 preclinical, N=2,797 phase I, N=4,121 phase II, N=826 phase III, N=797 launched. The center is the exact proportion and error bars are Wilson binomial 95% confidence intervals. See Figure S6 for the same analyses restricted to drugs with a single known target.*

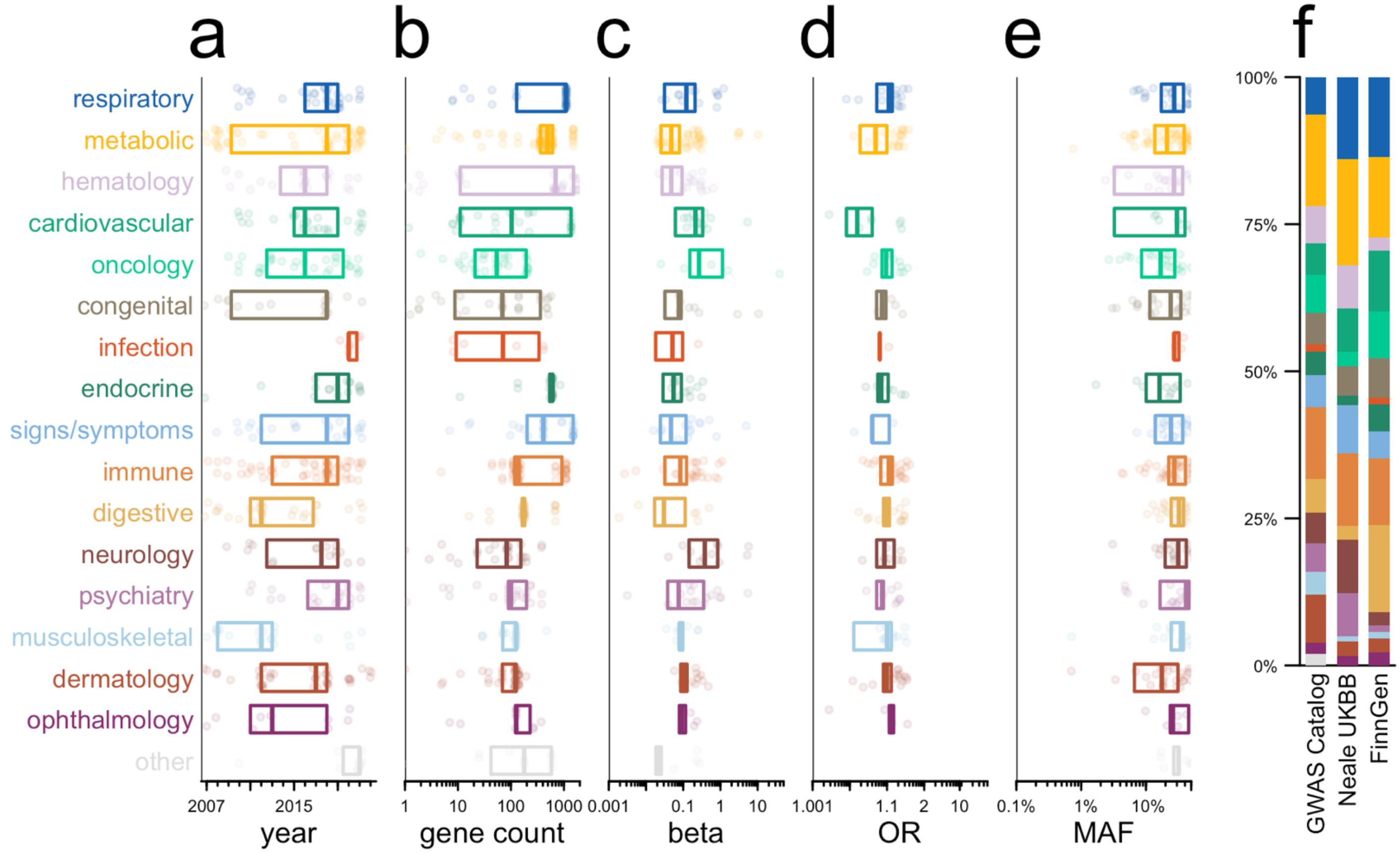

Figure S7. Confounding between therapy areas and properties of supporting genetic evidence (Extended Data Figure 6) in "one target only" mode. *In panels A-E, each point represents one GWAS Catalog-supported T-I pair in phase I through launched, and boxes represent medians and interquartile ranges (25th, 50th, and 75th percentile). Each panel A-E represents the cross-tabulation of therapy areas versus the properties examined in Fig. 1D. Kruskal-Wallis tests treat each variable as continuous, while chi-squared tests are applied to the discrete bins used in Figure 1D.* ***A****) Year of discovery, Kruskal-Wallis P = 1.2e-5, chi-squared P = 3.6e-9, N=445 target-indication-area (T-I-A) triplets;* ***B****) gene count, Kruskal-Wallis P = 2.1e-20, chi-squared P = 6.6e-30, N=517 T-I-A triplets;* ***C****) absolute beta, Kruskal-Wallis P = 1.7e-6, chi-squared P = 4.3e-3, N=301 T-I-A triplets;* ***D****) absolute odds ratio, Kruskal-Wallis P = 5.6e-5, chi-squared P = 6.6e-6, N=217 T-I-A triplets;* ***E****) minor allele frequency, Kruskal-Wallis P = 0.14, chi-squared P = 0.056, N=394 T-I-A triplets;* ***F****) Barplot of therapy areas of genetically supported T-I by source of GWAS data within OTG, chi-squared P = 0.0012.*

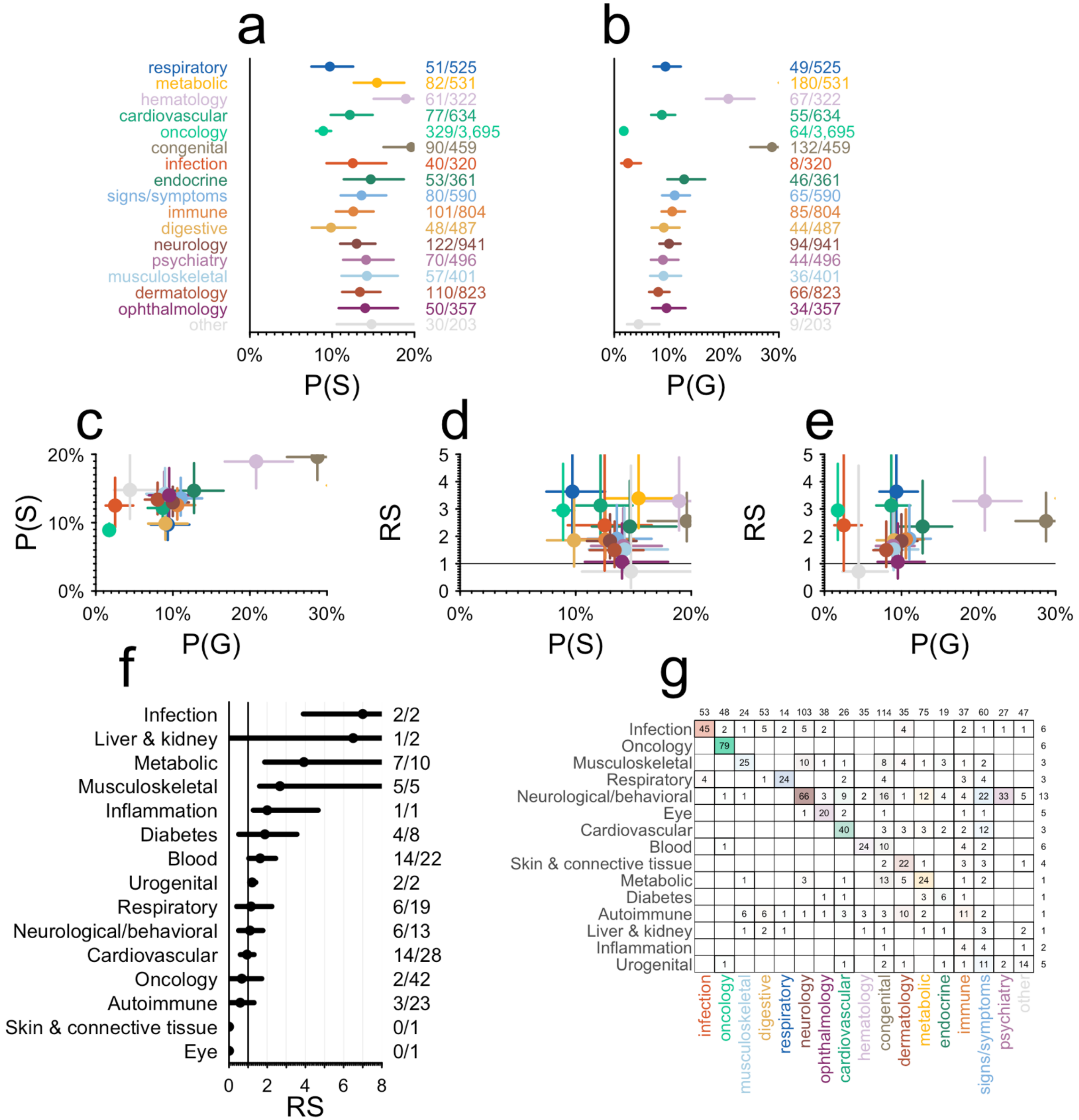

Figure S8. Further analyses of differences in relative success among therapy areas (Extended Data Figure 7) in "one target only" mode. A) *Probability of success, P(S), by therapy area, with Wilson 95% confidence intervals. The N shown at right indicates the number of launched T-I pairs (numerator) and number of T-I pairs reaching at least phase I (denominator). The center is the exact proportion and error bars are Wilson binomial 95% confidence intervals.* ***B)*** *Probability of genetic support, P(G), by therapy area, with Wilson 95% confidence intervals. The N shown at right indicates the number of genetically supported T-I pairs reaching at least phase I (numerator) and total number of T-I pairs reaching at least phase I (denominator). The center is the exact proportion and error bars are Wilson binomial 95% confidence intervals.* ***C)*** *P(S) vs. P(G),* ***D)*** *RS s. P(S), and* ***E)*** *RS vs. P(G) across therapy areas, with centers indicating point estimates and crosshairs representing 95% confidence intervals on both dimensions — Katz for RS and Wilson for P(G) and*

P(S). For A-E, total N=8,780 unique T-I pairs, but because some indications belong to >1 therapy area, N=10,498 target-indication-area (T-I-A) triples. ***F)*** *Re-analysis of RS (x axis) broken down by therapy area using data from supplementary table 6 of Nelson et al. 2015[5].* ***G)*** *Confusion matrix showing the categorization of unique drug indications into therapy areas in Nelson et al 2015 versus current. Note that the current categorization is based on each indication's position in the MeSH ontological tree and one indication can appear in >1 area, see Methods for details. Marginals along the top edge are the number of drug indications in each current therapy area that were absent from the 2015 dataset. Marginals along the right edge are the number of drug indications in each 2015 therapy area that are absent from the current dataset.*

a

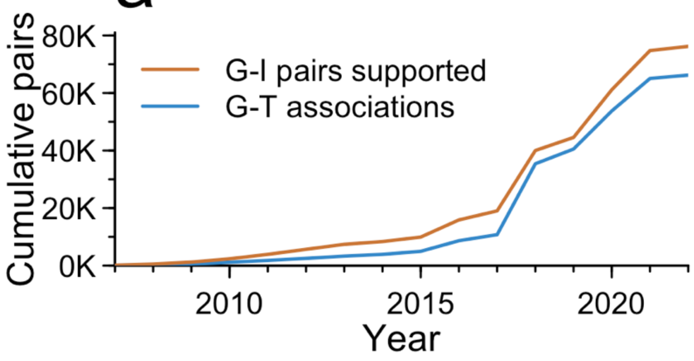

b

	all genes	predicted Ab tractable	predicted SM tractable	rhodopsin-like GPCRs	nuclear receptors	enzymes	ion channels	kinases
all areas	285/10424 **2.7%**	233/5292 **4.4%**	227/3213 **7.1%**	16/175 **9.1%**	8/34 **24%**	49/597 **8.2%**	15/210 **7.1%**	21/239 **8.8%**
respiratory	15/691 **2.2%**	14/368 **3.8%**	10/238 **4.2%**	2/6 **33%**	0/4 **0%**	1/42 **2.4%**	1/18 **5.6%**	0/10 **0%**
metabolic	76/3914 **1.9%**	62/2052 **3%**	60/1318 **4.6%**	4/52 **7.7%**	5/20 **25%**	8/249 **3.2%**	1/53 **1.9%**	2/104 **1.9%**
hematology	25/3072 **0.81%**	22/1514 **1.5%**	20/1046 **1.9%**	0/51 **0%**	0/13 **0%**	6/186 **3.2%**	0/35 **0%**	1/87 **1.1%**
cardiovascular	23/1706 **1.3%**	23/899 **2.6%**	20/578 **3.5%**	1/22 **4.5%**	0/10 **0%**	8/122 **6.6%**	4/48 **8.3%**	0/62 **0%**
oncology	76/2045 **3.7%**	58/949 **6.1%**	67/716 **9.4%**	4/26 **15%**	2/13 **15%**	16/130 **12%**	0/33 **0%**	15/79 **19%**
congenital	72/3555 **2%**	55/1912 **2.9%**	52/1311 **4%**	1/38 **2.6%**	1/19 **5.3%**	13/236 **5.5%**	1/80 **1.2%**	2/87 **2.3%**
infection	3/222 **1.4%**	3/129 **2.3%**	1/79 **1.3%**	0/4 **0%**	0/2 **0%**	0/13 **0%**	0/6 **0%**	0/2 **0%**
endocrine	23/2033 **1.1%**	20/1016 **2%**	20/667 **3%**	4/36 **11%**	2/22 **9.1%**	1/138 **0.72%**	1/40 **2.5%**	2/55 **3.6%**
signs/symptoms	29/2659 **1.1%**	25/1408 **1.8%**	25/842 **3%**	3/55 **5.5%**	2/19 **11%**	3/155 **1.9%**	6/73 **8.2%**	3/71 **4.2%**
immune	34/1566 **2.2%**	32/852 **3.8%**	22/545 **4%**	2/30 **6.7%**	0/4 **0%**	6/95 **6.3%**	0/19 **0%**	1/49 **2%**
digestive	20/1610 **1.2%**	16/871 **1.8%**	11/552 **2%**	0/33 **0%**	1/12 **8.3%**	2/95 **2.1%**	1/21 **4.8%**	1/51 **2%**
neurology	55/2810 **2%**	46/1531 **3%**	40/957 **4.2%**	2/44 **4.5%**	0/8 **0%**	8/164 **4.9%**	6/96 **6.2%**	2/67 **3%**
psychiatry	20/1663 **1.2%**	18/891 **2%**	19/524 **3.6%**	4/34 **12%**	0/9 **0%**	3/101 **3%**	4/55 **7.3%**	0/49 **0%**
musculoskeletal	21/1359 **1.5%**	20/735 **2.7%**	15/449 **3.3%**	1/15 **6.7%**	1/5 **20%**	1/82 **1.2%**	1/24 **4.2%**	3/42 **7.1%**
dermatology	31/1590 **1.9%**	27/869 **3.1%**	20/522 **3.8%**	1/18 **5.6%**	0/6 **0%**	5/106 **4.7%**	0/24 **0%**	2/45 **4.4%**
ophthalmology	19/1573 **1.2%**	14/888 **1.6%**	12/478 **2.5%**	1/16 **6.2%**	1/12 **8.3%**	2/87 **2.3%**	2/44 **4.5%**	0/43 **0%**
other	4/1186 **0.34%**	3/586 **0.51%**	3/363 **0.83%**	0/19 **0%**	1/7 **14%**	0/67 **0%**	0/28 **0%**	0/30 **0%**

Figure S9. Level of utilization of genetic support among targets (Extended Data Figure 8) in "one target only" mode. *As for Figure S3, but grouped by target instead of T-I pair. Thus, the denominator for each cell is the number of targets with at least one genetically supported indication, and each target counts towards the numerator if at least one genetically supported indication has reached phase I.*
