# Supplementary material for: Refining the impact of genetic evidence on clinical success
Source: Nature. 2024 Apr 17;629(8012):624–9. doi: 10.1038/s41586-024-07316-0 (PMC11096124; doi:10.1038/s41586-024-07316-0)
Supplement: Supplementary file 2 — Reporting Summary [file 41586_2024_7316_MOESM2_ESM.pdf]

nature portfolio

Corresponding author(s): Matthew R. Nelson

Last updated by author(s): 3/5/2024

Reporting Summary

Nature Portfolio wishes to improve the reproducibility of the work that we publish. This form provides structure for consistency and transparency in reporting. For further information on Nature Portfolio policies, see our Editorial Policies and the Editorial Policy Checklist.

Statistics

For all statistical analyses, confirm that the following items are present in the figure legend, table legend, main text, or Methods section.

n/a	Confirmed	
☐	☒	The exact sample size (*n*) for each experimental group/condition, given as a discrete number and unit of measurement
☒	☐	A statement on whether measurements were taken from distinct samples or whether the same sample was measured repeatedly
☐	☒	The statistical test(s) used AND whether they are one- or two-sided<br>*Only common tests should be described solely by name; describe more complex techniques in the Methods section.*
☐	☒	A description of all covariates tested
☐	☒	A description of any assumptions or corrections, such as tests of normality and adjustment for multiple comparisons
☐	☒	A full description of the statistical parameters including central tendency (e.g. means) or other basic estimates (e.g. regression coefficient) AND variation (e.g. standard deviation) or associated estimates of uncertainty (e.g. confidence intervals)
☐	☒	For null hypothesis testing, the test statistic (e.g. *F*, *t*, *r*) with confidence intervals, effect sizes, degrees of freedom and *P* value noted<br>*Give P values as exact values whenever suitable.*
☒	☐	For Bayesian analysis, information on the choice of priors and Markov chain Monte Carlo settings
☒	☐	For hierarchical and complex designs, identification of the appropriate level for tests and full reporting of outcomes
☐	☒	Estimates of effect sizes (e.g. Cohen's *d*, Pearson's *r*), indicating how they were calculated

Our web collection on statistics for biologists contains articles on many of the points above.

Software and code

Policy information about availability of computer code

Data collection	No software was used to collect data.
Data analysis	R 4.2.0 scripts used for data analysis have been made publicly available, see code availability statement. R package dependencies and the versions used are as follows: tidyverse_1.3.1, janitor_2.1.0, binom_1.1-1.1, glue_1.6.2, lawstat_3.4, weights_1.0.4, epitools_0.5-10.1, DescTools_0.99.45, openxlsx_4.2.5, optparse_1.7.1, MASS_7.3-56.

For manuscripts utilizing custom algorithms or software that are central to the research but not yet described in published literature, software must be made available to editors and reviewers. We strongly encourage code deposition in a community repository (e.g. GitHub). See the Nature Portfolio guidelines for submitting code & software for further information.

Data

Policy information about availability of data

All manuscripts must include a data availability statement. This statement should provide the following information, where applicable:

- Accession codes, unique identifiers, or web links for publicly available datasets
- A description of any restrictions on data availability
- For clinical datasets or third party data, please ensure that the statement adheres to our policy

Data availability. An analytical dataset is provided at https://github.com/ericminikel/genetic_support/ and is sufficient to reproduce all figures and statistics herein.

Human research participants

Policy information about studies involving human research participants and Sex and Gender in Research.

Reporting on sex and gender	N/A
Population characteristics	N/A
Recruitment	N/A
Ethics oversight	N/A

Note that full information on the approval of the study protocol must also be provided in the manuscript.

Field-specific reporting

Please select the one below that is the best fit for your research. If you are not sure, read the appropriate sections before making your selection.

☒ Life sciences ☐ Behavioural & social sciences ☐ Ecological, evolutionary & environmental sciences

For a reference copy of the document with all sections, see nature.com/documents/nr-reporting-summary-flat.pdf

Life sciences study design

All studies must disclose on these points even when the disclosure is negative.

Sample size	No pre-determined sample size was chosen; we used all available drug development data since 2000.
Data exclusions	We focused on the time period since 2000 because data are relatively complete for this time period.
Replication	Our study relies on retrospective analysis of drug development successes and failures worldwide since 2000, worldwide. Because human history has only occurred once, there is no opportunity for replication per se. Instead, to provide robustness, we performed each analysis under multiple different filters and conditions, for example, we re-ran the entire analysis restricting to only drugs with exactly one target, we examined the contribution of different genetic data sources, we varied our quantitative filtering thresholds, and so on. Core findings of our study were robust to all different conditions examined.
Randomization	Our study did not involve individual human or animal participants. Historical data on drug development successes and failures are inherently retrospective and cannot be randomized.
Blinding	Our study was retrospective in nature and there was no opportunity for prospective blinding. We note that many of the drug development programs analyzed here were undertaken before the discovery of relevant human genetic associations, and thus the pharmaceutical companies involved in these programs were in a sense "blinded", however, no formal blinding was undertaken.

Reporting for specific materials, systems and methods

We require information from authors about some types of materials, experimental systems and methods used in many studies. Here, indicate whether each material, system or method listed is relevant to your study. If you are not sure if a list item applies to your research, read the appropriate section before selecting a response.

Materials & experimental systems

n/a	Involved in the study	
☒	☐	Antibodies
☒	☐	Eukaryotic cell lines
☒	☐	Palaeontology and archaeology
☒	☐	Animals and other organisms
☒	☐	Clinical data
☒	☐	Dual use research of concern

Methods

n/a	Involved in the study	
☒	☐	ChIP-seq
☒	☐	Flow cytometry
☒	☐	MRI-based neuroimaging
